# p-Values and confidence intervals as compatibility measures: guidelines for interpreting statistical studies in clinical research

**DOI:** 10.1016/j.lansea.2025.100534

**Published:** 2025-01-28

**Authors:** Alessandro Rovetta, Luca Piretta, Mohammad Ali Mansournia

**Affiliations:** a Research and Disclosure, International Committee Against the Misuse of Statistical Significance (ICAMSS), Bovezzo, Italy; b Alimentary Science and Human Nutrition, ‘Campus Biomedico’ University, Rome, Italy; c Department of Epidemiology and Biostatistics, School of Public Health, Tehran University of Medical Sciences, Tehran, Iran

p-Values (p) are commonly misused in medical research to decide whether a result is significant (e.g., p ≤ 0.05) or not (e.g., p > 0.05). However, this oversimplification can translate into approving ineffective treatments or rejecting effective ones.^1^^,^^2^ Following the guidelines of the American Statistical Association and leading epidemiologists, this commentary reexamines the conclusions of previous papers published in *The Lancet Regional Health — Southeast Asia* using a more cautious approach, which better aligns with the interpretative nuances of clinical judgment.^1^, ^2^, ^3^, ^4^, ^5^, ^6^

A statistical test involves formulating a target hypothesis (e.g., the null hypothesis of zero effect) and then evaluating the agreement between experimental data and such hypothesis. The p-value helps answer the following question: How compatible (consistent, coherent) are the observed data with the prediction of the hypothesis under consideration? According to the chosen test, p-values close to 1 indicate high compatibility, while p-values close to 0 indicate low compatibility.^4^^,^^5^ A 95% ‘confidence’ interval (95% CI) can be employed to assess the consistency between data and several hypotheses about the effect instead of the sole null hypothesis. Indeed, a 95% CI is the range of all hypotheses with a degree of compatibility with the data greater than p = 0.05 (p > 0.05). Therefore, ‘confidence’ intervals should be renamed ‘compatibility’ intervals, as these do not express any confidence in our result but rather show its compatibility with various hypotheses.^4^^,^^5^ While a p-value alone tells nothing about the effect, a 95% CI provides a more clinically understandable range estimate by presenting values expressed at the level of data measurement (e.g., ‘mmHg’ for blood pressure). Thus, even though it is derived from the same mathematical framework as the p-value, a 95% CI offers substantial interpretative advantages and a more comprehensive information set.^d^

To illustrate, we consider a recent randomized study examining the impact of a nutritional intervention on reducing stunting in children at 24 months, which reports a ‘significant’ difference between intervention and control groups regarding weight.^7^ The researchers observed an average weight difference of 110 g in favor of the intervention group (3.1 kg vs. 3.0 kg) and a p-value for the ‘null hypothesis of exactly zero effect’ less than 0.05 (p = 0.01). Nonetheless, the key questions are: What is the clinical relevance of this 110 g difference? What is the consistency of such result with hypotheses of unimportant effects (e.g., non-exactly zero but still clinically small differences)?

To answer the first question, we note that the weight achieved in the intervention group (3.1 kg) is still far from the standard suggested by the World Health Organization (9.5 kg). Moreover, that outcome could result from other factors, including imperfect randomization or a sample size that is too small.^e^ To answer the second question, we note that the 95% CI—spanning from 0.03 kg to 0.20 kg – shows that the data are also reasonably compatible (p > 0.05) with hypotheses of very small differences (e.g., 0.031 kg, 0.032 kg, etc., as these values are contained within the 95% CI, Fig. 1). Thus, a more suitable conclusion would be: *‘These data are consistent with an average weight increase of 110 g due to nutritional intervention. However, since the observed difference is small and hypotheses of negligible differences also align well with the outcome, further investigations should clarify the clinical relevance of these findings.’*

**Fig. 1:**
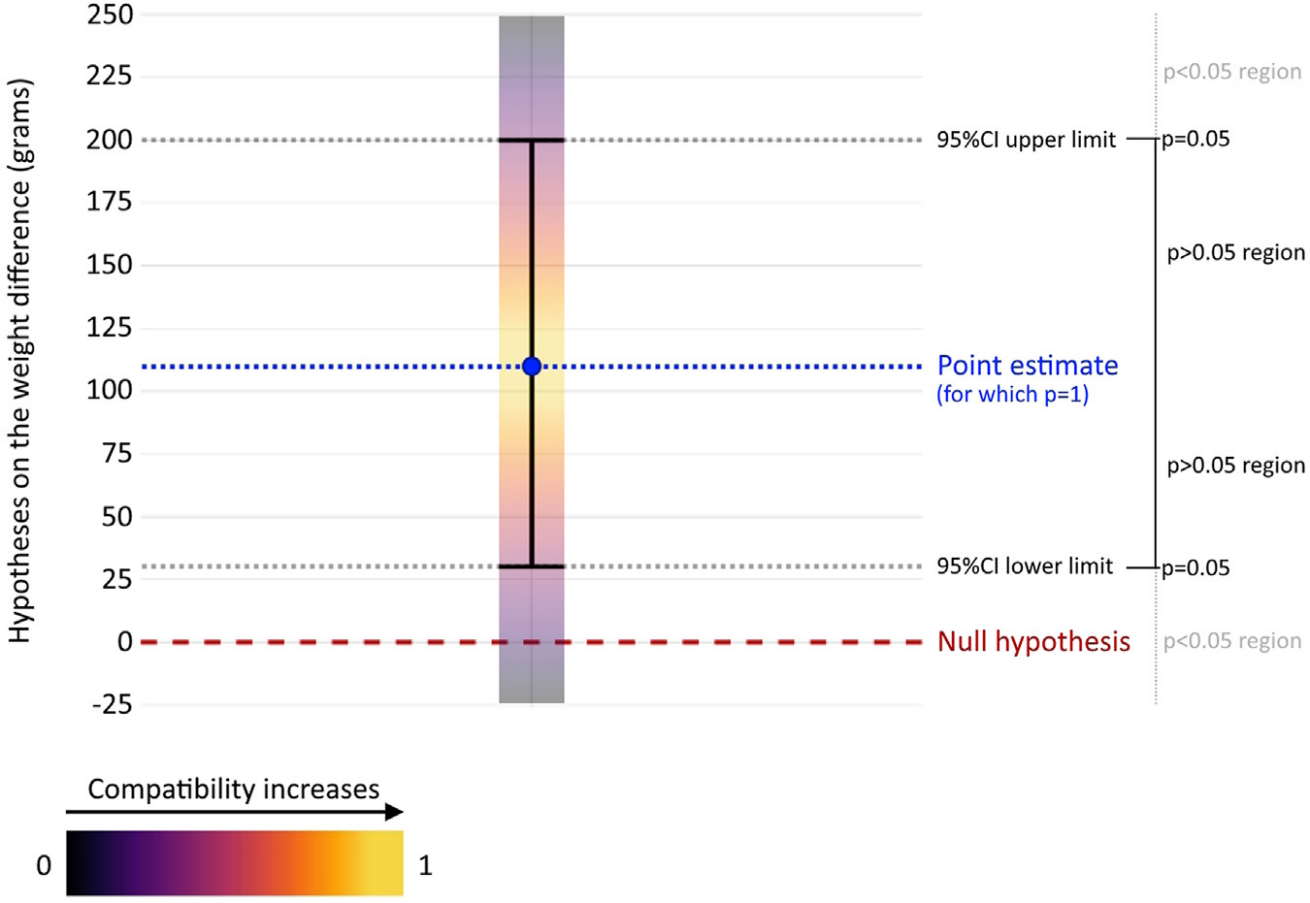
**Graphical representation of a 95% compatibility interval (CI) for the nutritional intervention example.** The vertical axis represents the hypotheses regarding the difference in weight between the intervention and control groups. As described by the color gradient, hypotheses close to the observed difference of 110 g (called ‘point estimate,’ represented by the blue dashed line) have p-values near 1, which means they are highly compatible with the experimental result (in fact, they are very close to the point estimate, see the yellowest region). Examples of hypotheses highly compatible with the observed difference are ‘100 g' and ‘125 g.’ As the hypotheses move away from the observed difference of 110 g, compatibility decreases (the p-value approaches 0, as represented by purple regions). Examples of hypotheses poorly compatible with the observed difference are '–25 g' and ‘250 g.’ The hypotheses at the interval limits (i.e., ‘30 g' and ‘200 g,’ see the gray dashed lines) have exactly p = 0.05; instead, those inside the interval have p > 0.05 while those outside have p < 0.05.

The same study also reports ‘no significant’ differences in the prevalence of wasting (−6.9%, p = 0.057, 95% CI from −14.1% to 0.3%) only because p > 0.05 for the null hypothesis of zero effect. Nonetheless, the observed result is not a zero difference but rather a 7-percentage-point difference. Simply, the 95% CI is so wide that it also includes clinically unimportant effects (e.g., 0%, 0.1%, 0.2%, etc.), suggesting high uncertainty in the estimate of the true effect. Therefore, a more cautious conclusion would be: *‘These findings are consistent with a 7% decrease in the prevalence of wasting in the intervention group. However, since both hypotheses of substantially larger and negligible effects reasonably agree with the data, future research is needed to confirm this result and assess its practical relevance.’* Ranges of effects (e.g., large, medium, small) should be defined before conducting the study based on prior knowledge about the cost-benefit ratio for the stakeholders, thus reducing post-hoc interpretations.

We emphasize that the compatibility approach does not invalidate the numerical results of previous studies: it is merely a criterion for drawing conclusions that are more grounded in clinical judgment rather than uncontextualized rules (like p ≤ 0.05 vs. p > 0.05).^8^, ^9^, ^10^ It also calls for caution when making health statements in light of the uncertainties that characterize medical sciences.^1^, ^2^, ^3^, ^4^, ^5^, ^6^^,^^8^, ^9^, ^10^ This request is corroborated by the inconsistencies concerning obesity-related papers; notably, over 40% of these studies explicitly mentioned that they considered ‘statistical significance’ only when p ≤ 0.05, implicitly referring to the null hypothesis of zero effect.^11^ In this context, the alternative term ‘compatibility’ suggests only a degree of agreement rather than significance, which instead requires solid multidisciplinary evidence.^1^, ^2^, ^3^, ^4^, ^5^, ^6^^,^^8^, ^9^, ^10^, ^11^ Compatibility can be better grasped by comparing the p-value to the probability of obtaining ‘s' consecutive heads in ‘s' fair coin tosses (S-value, see Appendix A).^4^^,^^9^^,^^10^^,^^12^

Our considerations align well with a recent viewpoint published in *The Lancet Regional Health–Southeast Asia*.^13^ We agree with Dr. Sterrantino that using the term ‘significant’ to describe results like p ≤ 0.05 is “misleading and inaccurate,” as it falsely suggests clinical significance.^f^ Dr. Sterrantino also stresses that the validity of statistical analyses depends on many background assumptions (e.g., hazard proportionality in Cox models) and processes. Among the latter is the ability to plan the research and ensure transparency in recognizing, presenting, and discussing limitations. We thus share the message that causality is not part of statistical calculations but rather “a narrative that we add to the statistical analysis and the scientific and statistical hypothesis that we are interested in.” This encompasses randomized studies, which remain susceptible to incorrect experimental procedures, misclassifications, biased samples, and misconduct. Accordingly, except in emergencies, health and policy recommendations should be formulated in dedicated systematic reviews with meta-analyses following decision analysis.^1^, ^2^, ^3^, ^4^, ^5^, ^6^^,^^8^, ^9^, ^10^^,^^12^^,^^13^

Finally, we note that the calculated p-value ‘p’ is the realization of a random variable ‘p’ (just as obtaining 'heads' after tossing a fair coin is the realization of the random variable ‘coin toss’).^14^^,^^15^ Even in desirable scenarios (e.g., high calculated power plus large sample sizes), the p-value is naturally subject to random fluctuations from sample to sample. This variability further highlights the inappropriateness of sharp decisional cutoffs in medicine (e.g., chance can easily produce p ≤ 0.05 in a study and p > 0.05 in its replication) and the need to focus instead on the contextual relevance of the measured effect. Efforts to constrain false positives and false negatives within fixed thresholds are generally undermined by unidentified sources of systematic errors, like long-term biases in the data-generation process (e.g., P-hacking, publication bias, wishful thinking, conflicts of interests) or variations in the parameter being estimated due to variations in the underlying causal context (e.g., changes in the treatment efficacy due to changes in population health behaviors). Conversely, the compatibility approach is far less sensitive to these issues, as the p-value represents only a single-sample descriptor of the agreement between data and the chosen statistical model rather than an inferential decision-making discriminant. In this regard, we acknowledge that our focus is on compatibility measures within a broader area, recognizing that statistical science encompasses methodological questions beyond the scope of this paper.^12^^,^^16^

## Contributors

AR: conceptualization, literature search, data curation, formal analysis, investigation, methodology, resources, software, visualization, writing–original draft, writing–review & editing.

LP: literature search, methodology, resources, validation.

MAM: literature search, methodology, resources, supervision, validation, review & editing.

All authors agreed on the submitted version.

## Declaration of interests

MAM is a statistical reviewer for The Lancet Group. We declare no other competing interests.

## Footnotes

d Tuples of p-values offer even more advantages as they show the exact p-value for many hypotheses of interest.^10^

e Such a small variation in weight could be related to a multitude of variables and covariates that may not be comparably captured in the two groups due to censoring-related selection bias, systematic errors in measurements, or insufficient degree of randomization.

f We clarify that it is very wrong to say that the p-value is the probability that the null hypothesis is true. Indeed, the hypothesis under examination (e.g., the null hypothesis) is one of the many conditions from which the p-value is mathematically derived. In other words, the chosen target hypothesis is assumed to be 100% true before conducting the test.
