# Supplementary material for: p-Values and confidence intervals as compatibility measures: guidelines for interpreting statistical studies in clinical research
Source: Lancet Reg Health Southeast Asia. 2025 Jan 28;33:100534. doi: 10.1016/j.lansea.2025.100534 (PMC11814670; doi:10.1016/j.lansea.2025.100534)
Supplement: Appendix A [file mmc1.docx]

**Appendix A**

Unfortunately, P-values exhibit counterintuitive behaviors. For instance, one might think that the difference in compatibility between two hypotheses with p=0.60 and p=0.55 is the same as the difference in compatibility between two hypotheses with p=0.06 and p=0.01, since in both cases the numerical gap between the pairs of P-values is 0.05 (i.e., 0.60−0.55=0.06−0.01=0.05). However, this is not the case. To understand why, let’s compare the P-value to the probability of getting 's' consecutive heads in 's' fair flips of a coin. Since the probability of success in a single flip is p=0.5, the probability of getting all heads in 's' flips is p=0.5^s^; it follows that s=−log_2_(p). For example, p=0.05 corresponds to s=−log_2_(0.05)=4.32; this means that the observed result is as surprising as about 4 consecutive heads in 4 fair tosses of a coin compared to what predicted by the target hypothesis.

We can now see that the S-value associated with p=0.65 is s=−log_2_(0.65)=0.62, while the S-value associated with p=0.60 is s=−log_2_(0.60)=0.74. This tells us that the information difference between the two P-values is unimportant (much less surprising than obtaining heads on a single fair coin flip, since 0.74−0.62=0.12<1). On the other hand, repeating the calculation for p=0.01 and p=0.06 yields s=6.64 and s=4.06, respectively, meaning that the information difference has higher importance compared to the previous one (it is more surprising than obtaining two consecutive heads in two fair flips of a coin, since 6.64−4.06=2.58>2).

S-values also show that dichotomizing compatibility (or significance) is a practice afflicted by elements of arbitrariness. For instance, a hypothesis with p=0.06 (included in the 95%CI) and one with p=0.04 (excluded from the 95%CI) have S-values of s=4.06 and s=4.64, respectively; thus, the information difference between them is negligible, i.e., less surprising than getting heads when flipping a coin once (since 4.64−4.06=0.58<1). In other words, there is no practical difference in the degree of compatibility between a hypothesis just outside the 95% CI and one just inside it.
